# Intrathecal adenosine enhances the antinociception of Xylazine in goats

**DOI:** 10.1186/s12917-022-03193-9

**Published:** 2022-03-17

**Authors:** Mahmoud M. Abouelfetouh, Eman Salah, Lingling Liu, Mingxing Ding, Yi Ding

**Affiliations:** 1 College of Veterinary Medicine, Huazhong Agricultural University, No.1, Shizishan Street, Hongshan District, Wuhan, 430070 Hubei Province China; 2 Department of Surgery, Radiology and Anaesthesiology, Faculty of Veterinary Medicine, Benha University, Moshtohor, Toukh, 13736 Egypt; 3 National Reference Laboratory of Veterinary Drug Residues (HZAU) and MAO Key Laboratory for Detection of Veterinary Drug Residues, Huazhong Agricultural University, Wuhan, 430070 Hubei Province China; 4 Department of Pharmacology, Faculty of Veterinary Medicine, Benha University, Moshtohor, Toukh, 13736 Egypt; 5 Clinical Veterinary Laboratory, College of Veterinary Medicine, Henan University of Animal Husbandry and Economy, Zhengzhou, 450046 Henan Province China

**Keywords:** Adenosine, Antinociception, Cardiorespiratory parameters, Xylazine, Goats

## Abstract

**Background:**

The role of adenosine (AD) in neuromodulation of nociceptive signaling at the level of the spinal cord has been established in both preclinical and clinical models. Recently, the signaling pathway that involves adenosine 5-monophosphate activated protein kinase has been reported to mediate the antinociceptive effects of xylazine (XYL). The objective of this study was to investigate the antinociceptive, cardiorespiratory and hematological effects of intrathecal administration of combined XYL-AD in goats as compared to XYL alone. Six clinically healthy adult goats weighing 25 ± 2 kg were randomly assigned to one of three groups in a cross-over design. Goats were sedated with XYL (0.05 mg/kg, IM) in all groups. Ten min later, 0.9% saline solution [SAL group], XYL (0.05 mg/kg) [XYL group] or a combination of XYL (0.05 mg/kg) and AD (2000 µg) [XYL-AD group] was injected intrathecally. Antinociception scores and both cardiorespiratory and hematological parameters were measured before XYL sedation and intrathecal injection (baseline), and at 5, 10, 15, 30, 60, 90, 120 and 150 min thereafter.

**Results:**

The XYL-AD group showed significantly earlier onset of antinociception [5 (5–7) min] than XYL [13 (12–14.25] min (*P* = 0.031). The duration of complete antinociception in goats that received XYL-AD was significantly longer (*P* = 0.031) than that received XYL alone [65 (58.75–66.25) and 47.5 (43.75–51.25) min, respectively]. In both XYL and XYL-AD groups, heart rate (HR), arterial blood pressure (SAP, MAP and DAP) were significantly decreased (*P* < 0.05) compared to the baseline. Compared to the SAL group, a statistically significant reduction in HR from 10 to 150 min (*P* < 0.05) was detected in the XYL group contrary to the XYL-AD group. Differences in the hematological parameters among different groups were insignificant.

**Conclusions:**

AD injected intrathecally interacts synergistically with XYL to promote antinociception in goats. This discovery supports the use of AD in combination with XYL in clinical trials.

## Background

Adenosine (AD) is a purine nucleoside molecule that is formed from the breakdown of adenosine triphosphate (ATP) [1]. Adenosine influences a variety of biological responses when it binds to four G-protein coupled adenosine receptors A_1_, A_2A_, A_2B_ and A_3._ These receptors are widely distributed throughout the body, but are especially concentrated within the cardiovascular, hemopoietic, and central nervous systems. Receptors can become activated by either endogenous or exogenous AD or its analogs [2, 3]. One of the many consequences of activation of spinal A_1_ and A_2_ receptors is the modulation of nociceptive signaling within the dorsal horn of the spinal cord [4–8]. Nociceptive input can be inhibited through several mechanisms, including presynaptic inhibition of excitatory neurotransmitters, hyperpolarization of spinal neurons by activation of K^+^ currents and inhibition of Ca^2+^ conductance [9–11].

Activation of A_1_ and A_2_ receptors also impacts the cardiovascular system. Binding of AD to A_1_ receptors inhibits catecholamine release, thereby slowing atrioventricular conduction whereas binding of AD to A_2A_ reverses the anti-adrenergic effect and subsequently increases cardiac contractility. Recent reports have demonstrated that AD has the potential to induce tachycardia by triggering sympathetic excitation as well as catecholamine release [12–15]. In addition, administration of AD via infusion has been reported to induce tachycardia, increased hemoglobin concentration and decreased blood pressure in pregnant ewes [16]. It is possible that the reported change in hemoglobin is due to activation of A_3_ receptors, which stimulates proliferation of precursor cells for erythrocytes, granulocytes, and macrophages. This would alter cellular components of the peripheral blood [17, 18].

Xylazine (XYL) is an α_2_ adrenergic receptor agonist that is commonly administered as a sedative and antinociceptive agent in veterinary practice. Xylazine mediates pain management when it binds to receptors that are located pre- and postsynaptically on the nociceptive neurons within the dorsal horn of the spinal cord. Binding of xylazine to these receptors triggers a reduced response to sympathetic activation or intravascular norepinephrine [19].

Recent efforts have concentrated on the adenosine 5-monophosphate activated protein kinase (AMPK) signaling pathway as a means of modulating the antinociceptive effect of XYL [20]. It has also been theorized that the antinociceptive effects of XYL might be magnified if XYL is administered in combination with other analgesic and anesthetic agents, including opioids [21], lidocaine [22] and ketamine [23]. Regional antinociception achieved by intrathecal and epidural injections is commonly used in small ruminants. Intrathecal administration allows analgesic agents to bypass the meningeal barriers to reach the dorsal horn of the spinal cord to impact their actions [24].

To our knowledge, no published articles have determined the effects of intrathecal administration of combined XYL-AD in goats. Our objective was to document the antinociceptive, cardiorespiratory and hematological effects of this combination. We hypothesized that AD would provide synergistic antinociceptive when administered in combination with XYL.

## Results

**Fig. 1:**
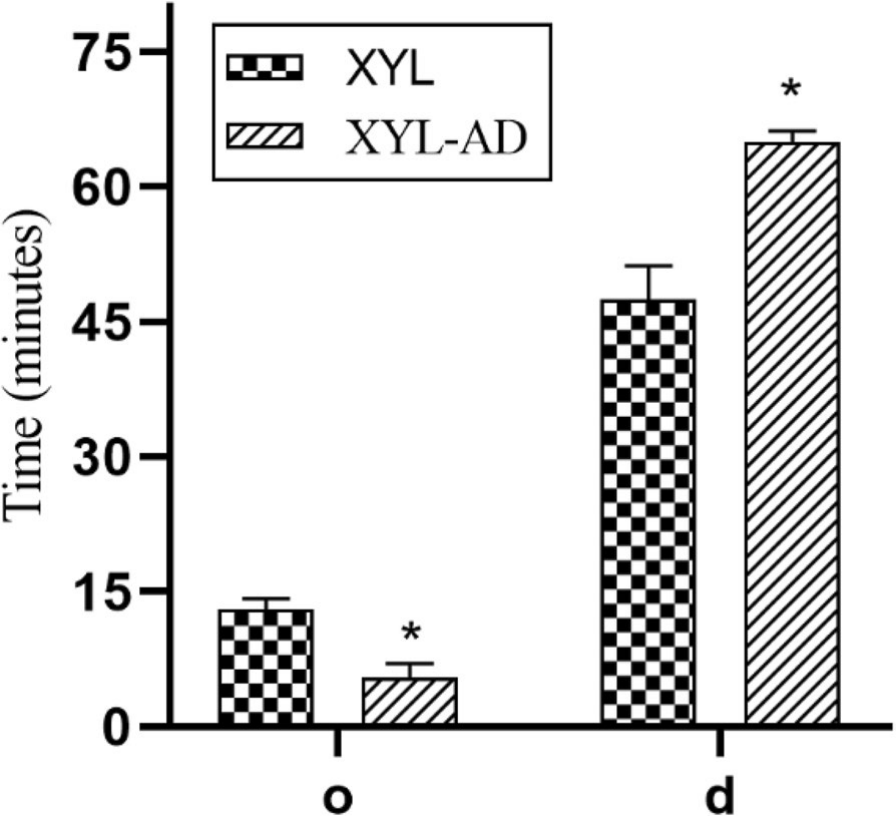
Onset (o) and duration (d) (median [IQR]) of complete antinociception for intrathecal xylazine (XYL) and xylazine-adenosine (XYL-AD). An asterisk denotes a significant difference between groups (*P* < 0.05)

**Fig. 2:**
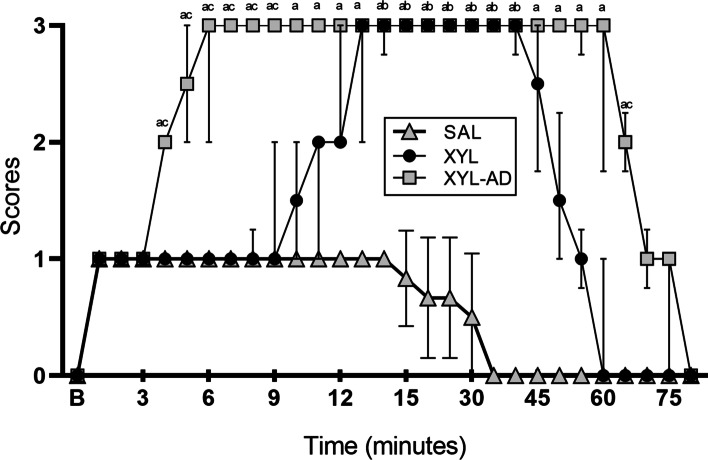
Antinociception scores (median [IQR]) before and after intrathecal saline (SAL), xylazine (XYL) and xylazine-adenosine (XYL-AD) in XYL-sedated goats. ^a^significantly different between the SAL and XYL-AD groups. ^b^significantly different between the SAL and XYL groups. ^c^significantly different between the XYL and XYL-AD groups (*P* < 0.05)

In this current study, intrathecal administration of XYL alone or combined with AD could induce complete antinociception of the umbilicus and caudal abdominal regions. The onset and duration of antinociception and antinociception scores were significantly different between the two groups. The XYL-AD group experienced significantly earlier onset of antinociception [ 5 (5–7) min] than the XYL group [ 13 (12–14.25) min (*P* = 0.031)]. The duration of complete antinociception (score 3) induced in the XYL-AD group was significantly longer (*P* = 0.031) than that induced in the XYL group [ 65 (58.75–66.25) and 47.5 (43.75–51.25) min, respectively] (Fig. 1). The antinociception scores were significantly higher in the XYL-AD group from 4 to 9 min and at 65 min (*P* < 0.05) compared to the XYL group. Compared to the SAL group, the XYL-AD group showed a significant difference from 4 to 65 min. So did the XYL group, from 14 to 40 min (*P* < 0.05) (Fig. 2).

**Fig. 3:**
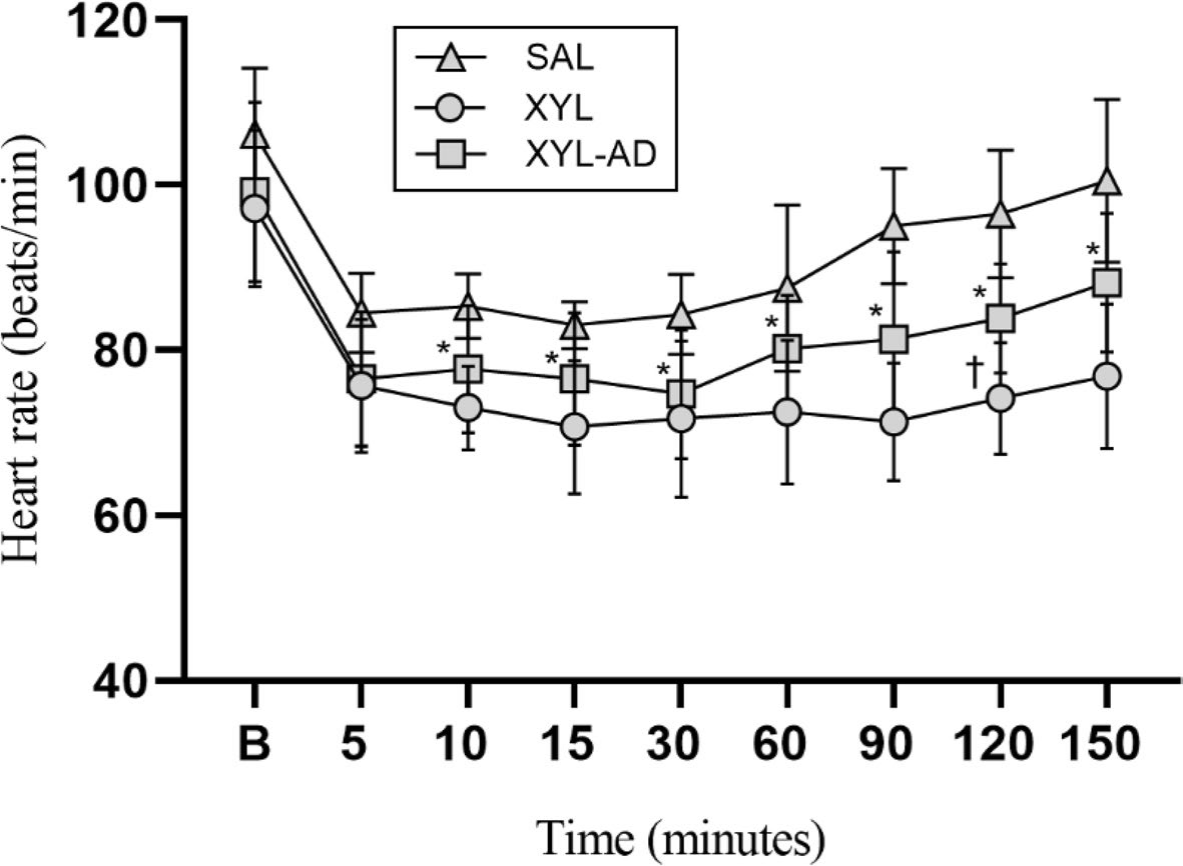
Mean ± S.D. of heart rate (HR) after intrathecal administration of saline (SAL), xylazine (XYL) and xylazine-adenosine (XYL-AD) in XYL-sedated goats. *significantly different between the SAL and XYL groups. † significantly different between the SAL and XYL-AD groups (*P* < 0.05)

In both the XYL and XYL-AD groups, heart rate (HR) significantly decreased (*P* < 0.05) between 5 to 90 min after intrathecal administration as compared to baseline. Compared to the SAL group, the XYL group exhibited a statistically significant reduction in HR from 10 to 150 min (Fig. 3). Systolic, mean, and diastolic arterial pressures (SAP, MAP and DAP) reduced significantly (*P* < 0.05) in both XYL and XYL-AD groups compared to the baseline and remained low for 120 min. Additionally, a significant decrease occurred in respiratory rate (RR) in both groups from 10 to 30 min and 15 to 30 min, respectively. There was non-significant change in hemoglobin oxygen saturation (SpO_2_) throughout the experimental period. Compared to the SAL group, the XYL-AD group showed a significant decrease in rectal temperature (RT) from 5 min, and the XYL group from 15 min through to completion of the experiment (150 min) (Table 1).

**Table 1:** Cardiorespiratory parameters at 5, 10, 15, 30, 60, 90, 120 and 150 min following intrathecal saline (SAL), xylazine (XYL) and xylazine- adenosine (XYL-AD) in XYL-sedated goats

Parameter	Group	Time after intrathecal administration (minutes)								
		**Base **^**b**^	**5**	**10**	**15**	**30**	**60**	**90**	**120**	**150**
**HR (beats/ min)**	**SAL**	106.1 ± 7.9	84.5 ± 4.8^a^ *P* = 0.003	85.3 ± 3.8^a^ *p* = 0.004	83 ± 3^a^ *p* = 0.001	84.3 ± 4.8^a^ *p* = 0.033	87.5 ± 10	95 ± 6.9	96.5 ± 7.7	100.5 ± 9.8
	**XYL**	97.1 ± 9.4	75.6 ± 8^a^ *p* = 0.005	73 ± 5^ab^ ^*a*^*p* = 0.002 ^b^*p* = 0.002	70.6 ± 8^ab^ ^*a*^*p* < 0.001 ^b^*p* = 0.027	71.6 ± 9.4^ab^ ^*a*^*p* = 0.002 ^b^*p* = 0.048	72.5 ± 8.6^ab^ ^*a*^*p* = 0.007 ^b^*p* = 0.049	71.3 ± 7.1^ab^ ^*a*^*p* = 0.001 ^b^*p* < 0.001	74.1 ± 6.7^ab^ ^*a*^*p* = 0.031 ^b^*p* < 0.001	76.8 ± 8.7^b^ ^b^*p* = 0.004
	**XYL-AD**	99.1 ± 10	76.5 ± 8.1^a^ *p* < 0.001	77.6 ± 7.6^a^ *p* = 0.002	76.5 ± 8^a^ *p* = 0.001	74.6 ± 7.7^a^ *p* < 0.001	80.1 ± 6.4^a^ *p* = 0.022	81.3 ± 10.5^a^ *p* < 0.001	83.8 ± 6.5^b^ ^b^*p* = 0.031	88.1 ± 8.4
**SAP (mmHg)**	**SAL**	118.5 ± 9.2	94.1 ± 7.1^a^ *p* = 0.006	98.8 ± 8.6^a^ *p* = 0.023	101 ± 11^a^ *p* = 0.016	110.3 ± 11.3	105.8 ± 10.6	107.1 ± 4.3	108.3 ± 9	116.8 ± 12
	**XYL**	118.8 ± 10.2	93.1 ± 8.8^a^ *p* = 0.007	87.6 ± 9.7^a^ *p* < 0.001	89.5 ± 12.1^a^ *p* = 0.003	86.8 ± 11^ab^ ^*a*^*p* < 0.001 ^b^*p* = 0.012	84.8 ± 7.9^ab^ ^a^*p* = 0.006 ^b^*p* = 0.009	89.3 ± 8.2^ab^ ^*a*^*p* = 0.018 ^b^*p* = 0.005	101.6 ± 11.9	113.1 ± 12.5
	**XYL-AD**	121 ± 10.5	88.8 ± 16.1^a^ *p* = 0.026	85.5 ± 11.8^a^ *p* = 0.006	79.1 ± 13^ab^ ^*a*^*p* = 0.004 ^b^*p* = 0.027	80.3 ± 14.3^ab^ ^*a*^*p* = 0.041 ^b^*p* = 0.07	86 ± 12.8^ab^ ^*a*^*p* = 0.038 ^b^*p* = 0.039	88.1 ± 12.8^ab^ ^*a*^*p* = 0.019 ^b^*p* = 0.006	95.3 ± 12.3^a^ *p* = 0.047	114.5 ± 11.6
**MAP (mmHg)**	**SAL**	87.1 ± 7.4^a^ *p* = 0.002	66.8 ± 3.5^a^ *p* = 0.014	71.6 ± 5^a^ *p* = 0.002	70 ± 4^a^ *p* = 0.026	73.5 ± 3^a^ *p* = 0.031	73.3 ± 2.5	75.8 ± 2	80.5 ± 4	83.5 ± 7.1
	**XYL**	85.6 ± 7.5	66 ± 1.5 *p* = 0.009	64 ± 6.3 ^a^ *p* = 0.006	62.8 ± 5.5^ab^ ^*a*^*p* = 0.001 ^b^*p* = 0.011	64.1 ± 4.9^ab^ ^*a*^*p* < 0.001 ^b^*p* = 0.011	63.6 ± 5.2^a^ *p* = 0.010	71.1 ± 5.2	78 ± 5.7	79.1 ± 5.4
	**XYL-AD**	86.3 ± 4.9	66 ± 6.8 *p* = 0.006	64.5 ± 5.1^a^ *p* = 0.001	61.1 ± 7.1^ab^ ^*a*^*p* = 0.002 ^b^*p* = 0.024	59.8 ± 8.6^ab^ ^*a*^*p* = 0.011 ^b^*p* = 0.009	63.5 ± 5.1^ab^ ^*a*^*p* = 0.006 ^b^*p* = 0.013	64.5 ± 6.1^ab^ ^*a*^*p* = 0.018 ^b^*p* = 0.031	72.8 ± 4.6 *p* = 0.019	81.5 ± 7.8
**DAP (mmHg)**	**SAL**	71.8 ± 7.7	53.5 ± 3.7^a^ *p* = 0.003	58.6 ± 4.8^a^ *p* = 0.044	54.8 ± 3.7^a^ *p* = 0.008	56 ± 3.2^a^ *p* = 0.044	59.1 ± 2.3^a^ *p* = 0.049	60.3 ± 2	66.8 ± 5.9	67.1 ± 5.7
	**XYL**	69.5 ± 7.3	52.6 ± 3.7^a^ *p* = 0.026	52.8 ± 4.9^a^ *p* = 0.020	49.8 ± 4.4 ^a^ *p* = 0.013	53.3 ± 3.5^a^ *p* = 0.012	53.3 ± 5^a^ *p* = 0.030	62.6 ± 5.1	66.5 ± 6.6	65 ± 7.3
	**XYL-AD**	68.6 ± 8	55.1 ± 4.9^a^ *p* = 0.004	54.3 ± 5.9^a^ *p* = 0.016	52.6 ± 7.2^a^ *p* = 0.046	50.1 ± 5.9^ab^ ^*a*^*p* = 0.010 ^b^*p* = 0.038	52.6 ± 4.5^a^ *p* = 0.002	60.3 ± 6.2	62.8 ± 7	65.5 ± 10.5
**SpO**_**2**_** (%)**	**SAL**	94.5 ± 2.2	95 ± 2.2	94.5 ± 2.2	94 ± 2.7	93.5 ± 2.4	95.3 ± 1.3	94 ± 3	95.3 ± 1.3	94.6 ± 2.4
	**XYL**	96.3 ± 1.5	93.8 ± 1.8	94.6 ± 1.6	93.6 ± 1.9	94.5 ± 1	95.1 ± 1.1	95.1 ± 1.7	95 ± 2	96.1 ± 1.3
	**XYL-AD**	95.8 ± 1.3	93.8 ± 1.6	94.5 ± 1.8	94.8 ± 1.4	94.5 ± 1.6	94.3 ± 1.5	95.3 ± 1	95.1 ± 2.4	95.6 ± 1
**RR (breaths/min)**	**SAL**	22.8 ± 4.5	19 ± 3.2^a^ *p* = 0.043	16.1 ± 2.1^a^ *p* = 0.036	15 ± 2^a^ *p* = 0.017	15.3 ± 2.1^a^ *p* = 0.014	17 ± 3	17.3 ± 2.6	18.6 ± 2.7	21.3 ± 3
	**XYL**	23 ± 4.5	17.1 ± 3.5	16.5 ± 3.2^a^ *p* = 0.012	14 ± 3.7^a^ *p* = 0.035	15.1 ± 3.1^a^ *p* = 0.048	19.1 ± 3.9	17.5 ± 3	20 ± 4	21 ± 3.8
	**XYL-AD**	23.1 ± 4.9	18.8 ± 2.2	19.1 ± 3.7	17.6 ± 3^a^ *p* = 0.050	16.3 ± 3.7^a^ *p* = 0.032	14.6 ± 3	19.1 ± 3.4	19.3 ± 3.7	21.3 ± 3.8
**RT ( °C)**	**SAL**	39.3 ± 0.12	39.2 ± 0.09	39.1 ± 0.09^a^ *p* = 0.002	39.1 ± 0.05^a^ *p* = 0.004	39.03 ± 0.05^a^ *p* < 0.001	38.9 ± 0.07^a^ *p* < 0.001	38.9 ± 0.05^a^ *p* < 0.001	38.7 ± 0.11^a^ *p* < 0.001	38.7 ± 0.08^a^ *p* < 0.001
	**XYL**	39.2 ± 0.05	39.1 ± 0.05	39 ± 0.05	38.9 ± 0.07^ab^ ^*a*^*p* < 0.001 ^b^*p* < 0.001	38.6 ± 0.07^ab^ ^*a*^*p* < 0.001 ^b^*p* < 0.001	37.8 ± 0.07^ab^ ^*a*^*p* < 0.001 ^b^*p* < 0.001	37.6 ± 0.08^ab^ ^*a*^*p* < 0.001 ^b^*p* < 0.001	37.5 ± 0.06^ab^ ^*a*^*p* < 0.001 ^b^*p* < 0.001	37.5 ± 0.04^ab^ ^*a*^*p* < 0.001 ^b^*p* < 0.001
	**XYL-AD**	39.2 ± 0.07	39.1 ± 0.07^b^ *p* = 0.048	39 ± 0.07^b^ *p* = 0.049	38.9 ± 0.07^ab^ ^*a*^*p* < 0.001 ^b^*p* < 0.001	38.6 ± 0.05^ab^ ^*a*^*p* < 0.001 ^b^*p* < 0.001	38.2 ± 0.05^ab^ ^*a*^*p* < 0.001 ^b^*p* < 0.001	37.9 ± 0.13^ab^ ^*a*^*p* < 0.001 ^b^*p* < 0.001	37.9 ± 0.13^ab^ ^*a*^*P* < 0.001 ^b^*p* < 0.001	38.1 ± 0.08^ab^ ^*a*^*p* < 0.001 ^b^*p* < 0.001

*HR* heart rate; *SAP* systolic arterial blood pressure; *MAP* mean arterial blood pressure; *DAP* diastolic arterial blood pressure; *SpO*_*2*_ hemoglobin oxygen saturation; *RR* respiratory rate; *RT* rectal temperature

Data is expressed as mean ± SD (*n* = 6)

^a^significantly different from the base value (*p* < 0.05) within each group; ^b^values taken before intramuscular xylazine and intrathecal injection

^b^significantly different from the SAL group at the same time point (*P* < 0.05)

In both XYL and XYL-AD groups, white blood count (WBC), lymphocyte %, red blood count (RBC), hemoglobin concentration (HGB), hematocrit (HCT) and mean corpuscular volume (MCV) were lower, while mean corpuscular hemoglobin (MCH), mean corpuscular hemoglobin concentration (MCHC) and granulocyte % were higher compared to the baseline during the experiment (Table 2). In both groups, these hematological fluctuations were observed anywhere from 10 to 120 min. They returned to near-baseline by the 150 min mark.

**Table 2:** Hematological parameters at 5, 10, 15, 30, 60, 90, 120 and 150 min following intrathecal saline (SAL), xylazine (XYL) and xylazine- adenosine (XYL-AD) in XYL-sedated goats

Parameter	Group	Time after intrathecal administration (minutes)								
		**Base **^**b**^	**5**	**10**	**15**	**30**	**60**	**90**	**120**	**150**
**WBC** **(× 10**^**9**^**/L)**	**SAL**	13.3 ± 2	12.5 ± 1.8	12.1 ± 1.7	12.2 ± 1.9	12.3 ± 1.7	12.8 ± 1.7	12.3 ± 1.6	12 ± 1.6	12.3 ± 2.2
	**XYL**	11.8 ± 1.4	11.5 ± 1.7	11.1 ± 1.4	10 ± 1.8	9.5 ± 1.6^ab^ ^*a*^*p* < 0.001 ^b^*p* = 0.046	10.1 ± 1.3^ab^ ^*a*^*p* = 0.019 ^b^*p* = 0.035	11 ± 1.5^a^ *P* = 0.019	11.5 ± 1.8	12 ± 1.4
	**XYL-AD**	12.3 ± 2.8	12.1 ± 2.5	11.5 ± 2.5	9.5 ± 2.3^a^ *p* = 0.001	8.8 ± 2.4^a^ *p* < 0.001	9.1 ± 2.5^ab^ ^*a*^*p* < 0.001 ^b^*p* = 0.043	10.3 ± 2.8^a^ *p* < 0.001	11.1 ± 2.9^a^ *p* = 0.004	12 ± 2.6
**Lymphocyte %**	**SAL**	50.8 ± 4.1	50.3 ± 3.8	49.7 ± 3.9	49.5 ± 4.1	49.6 ± 3.8^a^ *p* = 0.004	49 ± 4.3^a^ *p* = 0.019	49 ± 4	49.8 ± 3.5	50.1 ± 3.6
	**XYL**	50.8 ± 3.4	50.3 ± 3.3	49.8 ± 3.6	48.8 ± 3.6^a^ *p* = 0.003	47.8 ± 3.6^a^ *p* < 0.001	46.5 ± 2.5^a^ *p* = 0.001	47 ± 2.8^a^ *p* = 0.001	48.3 ± 2.4^a^ *p* = 0.030	49.1 ± 2.4
	**XYL-AD**	50 ± 4.2	49.6 ± 4.5	49 ± 4.4	48.2 ± 4.5^a^ *p* = 0.014	46.9 ± 4.4^a^ *p* = 0.002	45.4 ± 4.4^a^ *p* = 0.004	46.3 ± 4.5^a^ *p* = 0.009	47.1 ± 4.4^a^ *p* = 0.009	48 ± 4.2
**Granulocyte %**	**SAL**	37.6 ± 4.2	37.5 ± 4	39.8 ± 4.8	41.8 ± 3.7	43.8 ± 3.8^a^ *p* = 0.015	44.1 ± 3.9^a^ *p* = 0.003	44.8 ± 2.4^a^ *p* = 0.007	41.6 ± 3.4	39 ± 3
	**XYL**	37.8 ± 3.4	38.3 ± 3.6	40.6 ± 5.2	43.1 ± 4.1	47.5 ± 5.3^a^ *p* = 0.022	46.5 ± 4.3^a^ *p* = 0.011	48.5 ± 4.3^a^ *p* = 0.011	42.4 ± 5.3	41.6 ± 4.8
	**XYL-AD**	35.1 ± 2.9	35.5 ± 2.8	36.6 ± 1.9	37.6 ± 2.9^a^ *p* = 0.012	38.9 ± 3.7^a^ *p* = 0.014	41.6 ± 2.6^a^ *p* = 0.030	40.1 ± 2.5^b^ *p* = 0.024	39.3 ± 2.4	38.5 ± 2.6
**RBC** **(× 10**^**12**^**/L)**	**SAL**	20.1 ± 2.1	20 ± 2.3	19.6 ± 2.5	18.6 ± 2.1^a^	16.8 ± 1.8^a^ *p* = 0.019	17.1 ± 1.6^a^ *p* = 0.010	18 ± 1.6	18.1 ± 2	19.3 ± 1.9
	**XYL**	20 ± 1.4	20 ± 0.8	19.6 ± 1.5	17.8 ± 1.1^a^ *p* = 0.004	16.3 ± 1.2^a^ *p* = 0.002	17.3 ± 1.2^a^ *p* = 0.014	17.6 ± 0.8^a^ *p* = 0.024	18.3 ± 0.5	19.5 ± 1
	**XYL-AD**	19.3 ± 3	19 ± 2.6	18 ± 2.5	16.5 ± 2.4^a^ *p* = 0.001	15 ± 2.2^a^ *p* = 0.003	15.8 ± 2^a^ *p* = 0.011	16.8 ± 2.1^a^ *p* = 0.019	17.3 ± 2.5^a^ *p* = 0.013	18.5 ± 2.2
**HCT (%)**	**SAL**	29.5 ± 1.7	29.3 ± 2.3	28.1 ± 2.4	27.3 ± 2^a^ *p* = 0.004	27.1 ± 2.3^a^ *p* = 0.010	27 ± 2^a^ *p* = 0.014	27.8 ± 1.9^a^ *p* = 0.009	27.5 ± 1.7	28.6 ± 1.2
	**XYL**	29.6 ± 2.7	29.5 ± 3	27.3 ± 2.6 ^a^ *p* < 0.001	26 ± 2.2^a^ *p* < 0.001	25.6 ± 2.3^a^ *p* = 0.003	26 ± 1.8^a^ *p* = 0.006	26.8 ± 1.7^a^ *p* = 0.016	29 ± 2.3	29.5 ± 2.8
	**XYL-AD**	28.3 ± 2.7	27.6 ± 2.8	26.6 ± 2.8^a^ *p* = 0.002	25.6 ± 2.8^a^ *p* < 0.001	24.6 ± 2.6^a^ *p* < 0.001	25.3 ± 3.1^a^ *p* = 0.016	26.3 ± 3.1	27 ± 2.2	28.1 ± 2.5
**HGB (g/dL)**	**SAL**	9 ± 0.6	8.7 ± 0.8	8.4 ± 0.9	8 ± 0.8^a^ *p* = 0.005	8.1 ± 0.9^a^ *p* = 0.014	7.8 ± 0.9^a^ *p* = 0.007	7.9 ± 0.8^a^ *p* = 0.011	8.2 ± 0.7^a^ *p* = 0.020	8.5 ± 0.8
	**XYL**	9.2 ± 0.6	9 ± 0.8	8.7 ± 0.9	8.2 ± 0.8^a^ *p* = 0.007	7.8 ± 0.9^a^ *p* = 0.003	7.6 ± 0.7^a^ *p* = 0.003	7.8 ± 0.8^a^ *p* = 0.008	8.2 ± 0.9 ^a^ *p* = 0.019	8.6 ± 1
	**XYL-AD**	9.4 ± 0.5	9.3 ± 0.5	8.9 ± 0.9 ^a^ *p* = 0.013	8.5 ± 0.8^a^ *p* = 0.003	8.3 ± 0.5^a^ *p* = 0.002	8.2 ± 0.6^a^ *p* = 0.001	8.6 ± 0.6^a^ *p* = 0.004	8.6 ± 0.7^a^ *p* = 0.006	8.9 ± 0.6^a^ *p* = 0.010
**MCV (fL)**	**SAL**	22.5 ± 1.3	22.1 ± 1.7	21.3 ± 1.5	20 ± 1^a^ *p* = 0.004	19.5 ± 1^a^ *p* = 0.032	20 ± 1.4	21.3 ± 1.5	21.5 ± 1.2	22 ± 1.2
	**XYL**	22.3 ± 1.7	21.8 ± 2	21.6 ± 2.5	20.2 ± 1.5^a^ *p* = 0.004	19.8 ± 1.4^a^ *p* = 0.009	20.3 ± 1.6^a^ *p* = 0.016	21 ± 1.6	21.8 ± 1.7	22.1 ± 2
	**XYL-AD**	21.5 ± 3	21 ± 2.8	20.7 ± 2.8	19.9 ± 3.3^a^ *p* < 0.001	19.1 ± 3.4^a^ *p* < 0.001	19.5 ± 3.4^a^ *p* = 0.001	20.4 ± 3.2^a^ *p* < 0.001	21 ± 3.1	21.2 ± 2.8
**MCH (pg)**	**SAL**	7.8 ± 0.9	8 ± 0.8	7.8 ± 0.7	8.8 ± 1	9.9 ± 1^a^ *p* = 0.023	10 ± 0.8^a^ *p* = 0.017	10.4 ± 1.1^a^ *p* = 0.025	9.4 ± 0.9	8.4 ± 1
	**XYL**	7.5 ± 0.6	7.6 ± 0.8	8 ± 0.8	9.5 ± 1^a^ *p* = 0.001	10.3 ± 1^a^ *p* < 0.001	10.8 ± 1.2^a^ *p* < 0.001	10.6 ± 1.2^a^ *p* < 0.001	9.6 ± 1.2^a^ *p* = 0.003	8.6 ± 1.2^a^ *p* = 0.38
	**XYL-AD**	7 ± 1.3	7.1 ± 1.4	7.4 ± 1.1	8.8 ± 1.2^a^ *p* = 0.002	9.3 ± 1^a^ *p* = 0.003	10 ± 1.1^a^ *p* < 0.001	9.3 ± 0.9^a^ *p* = 0.002	8.5 ± 1^a^ *p* = 0.010	7.5 ± 1
**MCHC** **(g/ dL)**	**SAL**	45.8 ± 2.6	45.1 ± 3.1	45 ± 4	46.8 ± 2.8	48.1 ± 3.2	48.2 ± 2.8^a^ *p* = 0.004	48.8 ± 1.9^a^ *p* = 0.010	46.5 ± 2.1	46 ± 2.2
	**XYL**	44.5 ± 3.9	44 ± 4.1	45.8 ± 4.3	48.6 ± 4.3^a^ *p* = 0.002	50.6 ± 4.3^a^ *p* < 0.001	52.8 ± 3.8^a^ *p* < 0.001	50.8 ± 4.4^a^ *p* = 0.010	47.6 ± 4.1	45.3 ± 3.6
	**XYL-AD**	47.1 ± 3.7	46.6 ± 3.3	47.8 ± 4.4	50.8 ± 3.7^a^ *p* < 0.001	52.5 ± 3.9^a^ *p* < 0.001	54.3 ± 3.3^ab^ ^*a*^*p* < 0.001 ^b^*p* = 0.018	52.5 ± 3.5^a^ *p* = 0.007	50.6 ± 3.6^a^ *p* = 0.002	48.1 ± 3.7

*WBC* white blood count; *RBC* red blood count; *HCT* hematocrit; *HGB* hemoglobin; *MCV* mean corpuscular volume; *MCH* mean corpuscular hemoglobin; *MCHC* mean corpuscular hemoglobin concentration

Data is expressed as mean ± SD (*n* = 6)

^a^significantly different from the base value (*P* < 0.05) within each group; ^b^values taken before intramuscular xylazine and intrathecal injection

^b^significantly different from the SAL group at the same time point (*P* < 0.05)

## Discussion

Intrathecal administration of AD combined with XYL produced fast onset and prolonged duration of antinociception. Our findings demonstrated that AD acts synergistically with XYL to enhance antinociception. This study provides the first report about the effectiveness of combined XYL-AD administered intrathecally in goats. Previous studies have reported that intrathecal administration of AD increased antinociceptive threshold in rats that were exposed to acute thermal [25], and surgical pain [26]. Intrathecal administration of AD at a dose of 2000 µg was reported to reduce pain and produce a clinically relevant reduction of electrically induced temporal summation in humans [8, 27]. Additionally, a combination of intrathecal clonidine and AD has reportedly reduced hypersensitivity in patients with chronic regional pain syndrome [8]. A prior study also revealed that AD could potentiate the antinociceptive effect of acupuncture, which may improve the clinical application of integrative medicine [7]. XYL influences antinociception by binding to alpha-_2_ adrenergic receptors and exerting a local anesthetic-like action within the spinal cord [28, 29]. AD reduces neuronal excitability and nociceptive input in the spinal cord via activating A_1_ and A_3_ receptors, leading to modulation of Ca^2+^/K^+^ ions and γ aminobutyric acid (GABA) neurotransmission [30–32]. Recently, the AMPK signaling pathway has been reported to contribute in the central antinociceptive mechanism of XYL [20]. Following intrathecal administration of detomidine-lidocaine, the onset and duration of antinociception was 13.00 ± 1.89 min and duration for 66.25 ± 10.60 min, respectively [33]. Moreover, XYL at a dose of 0.1 mg/kg produced a complete antinociception with an onset of 9.5 ± 2.6 min and duration of 88.3 ± 15 min [34]. Compared to the previous studies, the combination of XYL at a dose of 0.05 mg/kg and AD produced earlier onset of antinociception, indicating AD act collaborativly with XYL to improve the antinociception.

The significant reduction in HR, SAP, MAP and DAP in both XYL and XYL-AD groups can be attributed to the depressant central and peripheral actions of XYL on sympathetic activity and catecholamines release [35]. Similar results after intrathecal or epidural administration of XYL have been reported in goats [23, 36]; sheep [37, 38]; cattle [39] and buffaloes [40]. A significant decrease in HR was observed in the XYL group, not in the XYL-AD group, as compared to the SAL group. This finding could be explained due to activation of AD A_2_ receptors that increase cardiac contractility directly or indirectly via attenuating antiadrenergic effect mediated by AD A_1_ receptors [41]. Previous studies reported that AD could elicit tachycardia in conscious man [13], and dogs [15]. Activation of central sympathetic tone might be also implicated in the AD-induced tachycardia [13]. Furthermore, AD infusion resulted in twofold increase in plasma circulating norepinephrine [42]. In this current study, AD could be slowly released into the circulation following intrathecal administration, so it could exert its systemic action at the level of the spinal cord and periphery. RR and RT were decreased in both XYL and XYL-AD groups and non-significant difference detected between the two groups. XYL administration has been reported to induce depression in respiratory [43] and thermoregulatory centers as well as reduction in the metabolic rate due to profound sedation and muscle relaxation [36]. There were no evidence of respiratory depression and /or hypothermia associated with AD administration [12, 16].

Both XYL and XYL-AD groups experienced significant alterations in RBC, WBC, lymphocyte %, HCT, HGB, MCV and granulocyte %, MCH and MCHC compared to the baseline. However, differences between the XYL and XYL-AD groups were insignificant. AD has not been reported to have a hematological influence [2], indicating XYL has been implicated in most changes in the peripheral blood constituents. These findings coincide with other studies reporting the hematological effect of systemic and epidural XYL administration in goats and horses [44–46]. Sequestration of blood in the spleen and other reservoir sites like liver, muscle, and skin as a result of decreased sympathetic outflow following XYL administration could be attributed to the decrease in RBC, HCT and HGB [47, 48]. Furthermore, XYL sedation has been reported to decrease RBC, WBC, MCV, HCT and HGB [36, 44], which might be explained by xylazine-induced hemolysis and hemodilution caused by fluid displacement from the lumen into the blood circulation. Alpha_2_-adrenergic agonists have been suggested to activate pulmonary intravascular macrophages and stimulate a series of inflammatory events resulting in recruitment of leukocytes in the lung circulation [49, 50]. This mechanism might be implicated in a decrease in WBC within the peripheral circulation. Moreover, XYL immobilization may induce a stressful condition, which could trigger adrenocortical function and glucocorticoid (GC) release [51]. The GC could provoke suppression of lymphoid tissues, resulting in decreased proliferation and apoptosis of lymphocytes. Moreover, XYL has a direct immunosuppressive effect on spleen via inhibiting splenocytes proliferation as well as inducing lymphocyte death [52]. Even though WBC and lymphocytes were significantly decreased, granulocytes (polymorphonuclear leukocytes; PMNs) were significantly increased [53]. The XYL itself and/or the induced GC may be attributed to the increase in circulatory PMNs via several biologic effects, including decreased margination of PMNs, delayed extravasation of PMNs into tissue, delayed rate of apoptosis and the release of immature neutrophils from the bone marrow into the circulation [54, 55]. The effects of XYL on blood cellular indices are at times contradictory and the exact mechanisms that underlie the changes in parameters remain undetermined.

The small sample size in this study may have limited the value of observations as a true representation of the goat population. In addition, a comprehensive evaluation would be provided if sedation score data was available in this study, but the main objective of this study was to document the antinociceptive, cardiorespiratory and hematological effects of intrathecal XYL (0.05 mg/kg) and AD (2000 µg) combination in goats. Moreover, additional studies were needed to explore the effect of intrathecal AD alone as well as explain the antinociceptive synergism of the XYL and AD.

## Conclusion

Intrathecal administration of AD (2000 µg) combined with XYL (0.05 mg/kg) enhances antinociception in goats. The onset of antinociception was faster, and the duration was longer as compared with intrathecal administration of XYL alone. Therefore, our data support using XYL-AD in clinical trials in goats to improve delivery and onset of antinociception.

## Material and methods

### Animals

Six clinically healthy adult female goats between the ages of 14.3 ± 0.7 months and weighing 25 ± 2 kg were included in this study. For the purposes of this study, a clinically healthy animal was defined as one that underwent comprehensive physical examination and tested within the normal reference ranges for complete blood count and biochemical profile. The goats were purchased locally and brought into experimental research unit one week prior to the experiment for daily acclimatization to handling and the environmental design. The animals were fed maize, wheat bran and alfalfa hay and gained free access to water. Goats were denied access to food or water for 8 h prior to the experiment. All experiments were carried out in the morning. At the conclusion of the study, goats were transferred to the Veterinary Teaching Farm to be maintained by the College of Veterinary Medicine, Huazhong Agricultural University. This study was approved by the Animal Experimental Ethical Inspection of Laboratory Animal Center, Huazhong Agricultural University (ID number: HAZUGO-2021–0001).

### Study design

**Fig. 4:**
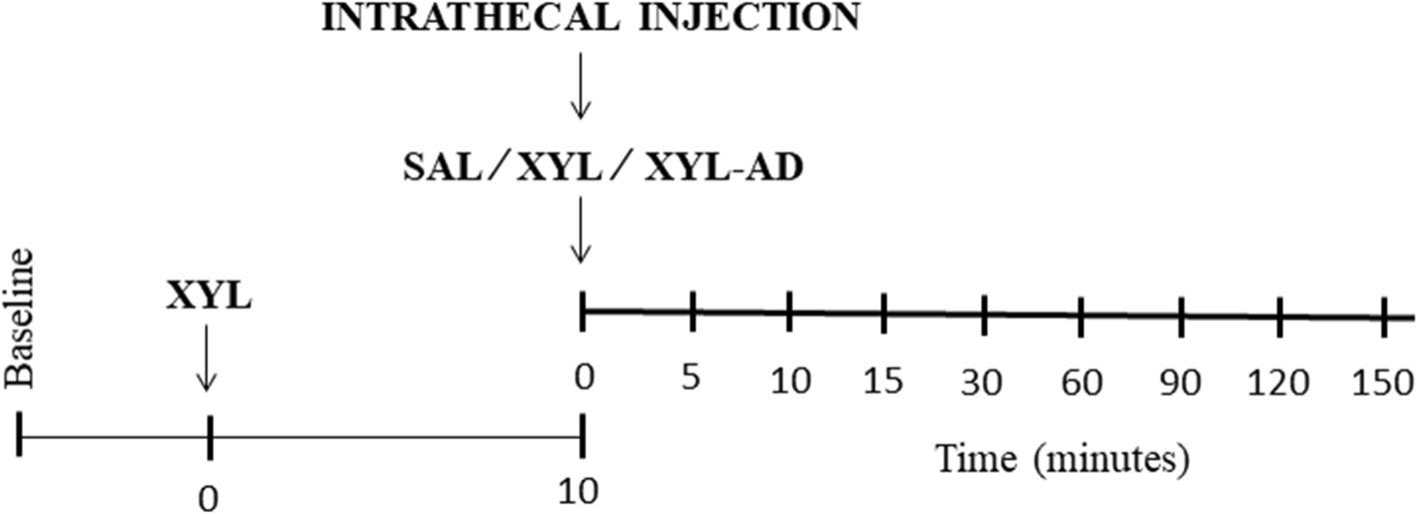
Study timeline illustrating the time of intramuscular xylazine (XYL) injection and intrathecal injection of 0.9% saline solution (SAL) and XYL and XYL- adenosine (AD) combination (XYL-AD). The data collection time points were at baseline and at 5, 10, 15, 30, 60, 90, 120 and 150 min after intrathecal injection

This experimental study made use of prospective, randomized crossover design. Goats were randomly assigned to one of three groups using a computer program (www.randomizer.org). Goats were sedated with XYL (0.05 mg/kg, IM; Xylaject 2%, Adwia, Egypt) in all groups. Ten min later, 0.9% saline solution (SAL group), XYL (0.05 mg/kg) (XYL group) or a combination of XYL (0.05 mg/kg) and AD (2000 µg/animal; Adenocor® 3 mg/mL; Sanofi, UK) (XYL-AD group) was injected intrathecally with a 7-day washout period. The agent(s) that were administered into each group were diluted in 3 mL saline (approx. 1 mL/ 7.5 kg). The size of needle used for intrathecal injection was 20 G and 3.5 cm in length. The lumbosacral region was clipped and prepared aseptically using betadine antiseptic solution (Betadine®; Mundipharma Pharmaceuticals Ltd). The skin and subcutaneous tissue over the lumbosacral region was infiltrated with Debocaine 2% (Sigma-Tec Pharmaceutical Industries). Following XYL sedation, the goats were positioned in right lateral recumbency for intrathecal injection. The needle was pointed at the level of the lumbosacral junction along the median line and directed into the spinal cord. The subarachnoid space was verified by free flow of cerebrospinal fluid through the hub of the needle. Antinociception scores and cardiorespiratory and hematological parameters were measured before XYL sedation and intrathecal injection (baseline), and at 5, 10, 15, 30, 60, 90, 120 and 150 min after intrathecal injection (Fig. 4).

### Antinociception scores measurement

An examiner who was blinded to drug treatments assessed the onset and duration of antinociception using pinprick needle stimulation at the level of umbilicus (2–4 cm from the midline at the level of umbilical stump) and caudal abdomen (2–4 cm from the midline midway between the umbilicus and pubic symphysis) using a modified scoring system of 0–3 (Appendix) [34]. In this current study, intrathecal injection was performed with the animal in the right lateral recumbency. Because the injected drugs tend to affect the spinal nerves suppling the downside, we tested for antinociception in the right umbilicus and caudal abdominal regions. A sum of 3 pinpricks using 23 g needle was applied through the skin, subcutaneous and muscular layers so that at each layer the antinociceptive response could be assessed. Purposeful reactions of head, neck, trunk, or limbs was defined as positive nociceptive reactions [56]. Following intrathecal injection, pinprick testing was done at 1-min intervals till occurrence of the onset of complete antinociception, and then at 5-min intervals until sensation was restored. The onset of antinociception was considered to be the time from intrathecal injection to complete loss of sensation (score 3), while the time from the onset to regain a moderate level of sensation (score 2) was defined as the duration of antinociception.

### Cardiorespiratory parameters and rectal temperature

HR (beats/min), non-invasive arterial blood pressures (SAP, MAP and DAP; mmHg), SpO_2_ (%), RR (breaths/min) as well as RT (°C) were measured using a multi-parameter 3-lead electrocardiogram (ECG) patient monitor (Mindray MEC-1200 Vet, Louisiana, US). In order to monitor blood pressure, an appropriately sized cuff (width was about 40% of the circumference of the limb) was positioned around the left metacarpal artery with the proximal end of the cuff 2.5 cm distal to the carpus [57].

### Hematological parameters

Blood samples (2 mL) were obtained from the jugular vein using single-use syringes and immediately placed in 3-mL glass tubes containing the anticoagulant ethyldiaminetetraacetic acid (EDTA). The blood indices, including WBC (× 10^9^/L), lymphocytes %, granulocytes %, RBC (× 10^12^/L), HCT (%), HGB (g/dL), MCV (fL), MCH (pg) and MCHC (g/dL) were determined using automated haematology analyzer (Mindary, BC-2800 Vet, Shenzhen, P.R.China).

### Statistical analysis

Statistical tests were performed using GraphPad Prism software version 8.0. The cardiorespiratory and hematological data were expressed as mean ± standard deviation (SD). The Kolmogorov–Smirnov test was used to confirm the normal distribution of data. Two-way ANOVA for repeated measures was used with Turkey's post-hoc test to compare variables between groups. One-way ANOVA was used with Dunnett's post-hoc test to compare variables within each group. Data of the antinociception scores, onset, and duration of antinociception were expressed as median and interquartile range [IQR] and compared using Friedman test with Dunn's post-hoc test. The differences were considered significant at *P* < 0.05.

## Data Availability

The data presented in this study are available on request from the corresponding author.

## Appendix

Table 3

**Table 3:** Antinociception scoring system

Score	Description
0	None: Presence of pain sensation in response to skin pinprick
1	Mild: Absence of pain sensation in response to skin pinprick
2	Moderate: Absence of pain sensation in response to subcutaneous pinprick
3	Complete: Absence of pain sensation in response to muscle pinprick

Modified after DeRossi et al. 2002

## Abbreviations

AD: Adenosine
ARs: Adenosine receptors
AMPK: Adenosine 5-monophosphate activated protein kinase
ATP: Adenosine triphosphate
DAP: Diastolic blood pressure
ECG: Electrocardiogram
EDTA: Ethyldiaminetetraacetic acid
HCT: Hematocrit
HGB: Hemoglobin concentration
HR: Heart rate
GABA: Gamma aminobutyric acid
GC: Glucocorticoid
MAP: Mean blood pressure
MCH: Mean corpuscular hemoglobin
MCV: Mean corpuscular volume
MCHC: Mean corpuscular hemoglobin concentration
PMNs: Polymorphonuclear leukocytes
RBC: Red blood count
RR: Respiratory rate
RT: Rectal temperature
SAL: Saline
SAP: Systolic blood pressure
SpO_2_: Hemoglobin oxygen saturation
WBC: White blood count
XYL: Xylazine
